# Record of Meteorological Conditions at Buffalo, for June, 1855

**Published:** 1855-08

**Authors:** Sanford B. Hunt


					﻿ART. VII. — Record of Meteorological Conditions at Buffalo, for June,
1855. By Sanford B. Hunt, M. D.
s	I
M	p_,	00
2 £ 2 1 £
«	"3	5	aS 1x3
• g	«	«	-g
co	-W	S	2
<i)	co	Js	fl
rs	£	co	£
.________________________O_________________________ Q____________________g____________________________£________
•gj’njwadaidj; |
io aiJu-tM ismjxA o t'® co tc -< co CM co cp ro — upt'coo<fl'#tom®mmot'01t't'io >'■
________ kJ|—'—■_________.___h__________—__	<m —>	—<	r-t —,	c-
•a’ii	x> t o	-ittco-'OOiOoi-- c - x t ® ~ - r. r. cc - r.	TSo"
nrrTTnTT	T.nir.-	J® CO	CO UP CP —I—< 71 Cl t > C. 4- -' ®1 -f -* _I- ® Cl I' PP PP®"!^ t,	<M
-JtmtlH	u«d[\i	®x®	t-t'<-, OlOOOOaOr^t-'t^ODOOClt'-OOTOOt^OOOOCSOOt-'aOQOOOt^	loo
!*	.ifrrr.T cd o a> o o co cd o o”o’co"CP PO CO CO cd"O’CO O 'X> O CPK o co o <o o o up
4«I°d	. co ®	® o; to co to -r r-i m	r- co o id	io lo co	co ® cs	® ®	h u-.	-r -r «	pi
5 Maj ueaw o ci cd	cd ad cd ci ci ci cd-rj cd	cdcciod	cd-4	cd cd cd	co ci-2 o cd	oo od cd	co
‘-O co ’i*	T -^ in -c ~ )-O I.o -C ’f iC IO i.O	iO CO i.o	in 1O to	CO CO	to	CC: tO	CO	X co	t'	UP
.,,,,,„	I -r co	cococococo co to co co to	coco	co	co	co’cn	co	co
dinjB	I co CO	. sD CO CO CO CO .COCOCOCOCO	. . .COCO	CO .	.	.CO	co CO	CO	UP
-jaaiusx uBjpjl up t~4 -4	co t-4 a -d co’ ad o co tc cd od od	4-4 co	o a:‘ ci	t-4 ci	*4	co’ od	co	up	ci	ci
_____________to up up	up up co up up up co up up u» up up	co co co	co up c—	to co	co	co co	t~-	i- S-	t-	co
o > co	o. (' -i^r-t'cocOTj’V’tMOuP 0-1 c: ®	o o I'	xc;	I' o r-	M c.o	it^
•	•.CatntmniTb5< )2 S?	^ oo co 00	of t-co-t<	O! o co	ao t~-f	to-«	10t'<'co co	.0
u	‘’■■w.lLUUHjao ao 00 00-jo op ao ao ao 00	□□ ao cp	00 00 co	00 00 op	<op ctp	00 00 ao	00 00 t~-	joo
= ‘lino t nan . *5 l® .l® . , co co cp. cp . ~ cnc; . .cocoTj<coi-<™oo(MupupTf<up co
o	.a- tj> oco o o o cp t^’up o cd o <oi ci od ci t4up cd ci ci r4 00 ci oo od cd up’
K • tx_______tfl	ry tfl lQ tfl	L.D
I
•	** •amrdmaT...............................................................................................*
_______________1.!^ 1.0 O L.Q IQ to lQ 1.^ 1Q Ip IQ to if) CD if) if) C'. CD tD *D to b-	tO
.W MJ
O	02 > 02 £ <Z2 ? 02	02020202 02 02
	—i~^cd-4oo-d<cdcdcicdTj<cd	—i c-5 co’ o pj -)<______________
g________________________________________________________________________________s'pnr>[3joi,uiv''Oi'Occocncoooco?i_0000000	1
os	U"> 22 5° S? 2 S2	to">d”cdci’—1 r4"<d""iS^d’up"up4rp-^7^5-IxrF4oi’t'' ci ci —•	-4
H	•ifnnimnrr V S !Z £2 E 5 17 00	15 00 01	0 00 ■* **	‘® *® s®	®> G* & « « up t"
COQOi'--Ot~-OOOCC'-C'-OOl'-t-t~-CPt'-CPUPUPCPOOCOOOt'-CPt— t-COt-t'-t'- r-
® o	.	—4
8 'JUIOJ Man .	.	. t© tO .	. O'! CO co Jt- CO CO CD ■—!	. COUPTftQOUPTjtMCOfM —‘CO tJ4
2	’ ^^*t^oocoot>-THO^cdciup-n<cico’i-Jupupo'upudupcdTi<’-Tiicir7o’o’cd od
•	d ________CO UP TF t-*4 up UP UP UP UP UP -Q4 t^4 UP UP UP CO UP UP CO UP CO CO CO CO CO CO CO ("• I— t ' up
<T ►»	~	“	'	' CO
. " •gjnidaia t. • ............................................................................................... —!
n.	- o*	®	® ci	o in ® up ?i ci tc u. -t ct	tn m m	pi H	j.	pi f, c; co 0	o c; ci	t-
t~~ CO	UP	UP to	to 1.0 UP to CO UP UP CO COCO	•Qt'-4-	CC CO	t~ I- CO t~ f t—	00 r^oo__ co
C§g°r	02 02	02 02	GQ 02 02	02	02	02	02 02 02 02	02 02 02’
*-< O J> .............................................
___________cd ci tJ cp cj t)4‘ Tji cd -« -4 cd cd	i?i cd ci cd ci cd cd
spn°[gjot^tuy ooopupi-te-ococoocpooo	ocooupooo
opt^cpcp-^cNCPCiC'Tf—ict'MapcDcoortt'Ortt'Oinr.aocpoi	c-
. •£>TOiuinh 5 !,- ■? cn ® cn x ?! t, _n_p „ ci c: c-i cp i.p x> c <' - o -t o u. ci o ® cc o	up
c.	. “'ll ® ® ® ® <0 ® cp ® ® o t't' X ® cp m® o o ® ® cpo o o x cp ® r® ,®	go
. 'inioj Marr •	.2*5'®.M'I .CPClr-tuPcncOT-iT^t^uPUP . C?t-p CO .CO-^OOCPT^OO O
o -t.d. Ur^ot^TftT^cicdoodi^ciTjit^cicdcdco’cit^THapcicicdoocdcdtdcd up
tao	co up -p4 -j4 Tj< up up up T-f up -t Tf -j4 up up u-p up tt to up u-p co no co co co co co co t '	up
&	co
t Mtu.duwx 'd-^<‘o’-4t4odup‘cdcdap-4up'^odupcpcic3PTHUpcdcd'd'cdcit4t4cdciod o’
c-- _________I co up up up up up UP UP UP up up up up up up co co up co up co co CO CO CO CO CO t— co
3 s7i3*
o § g o ■%	02 02	02 02 ®	02’ 02 02	02 02 02	02	02	02 02	02 02	02
Q r*	................................... .........................
___________eQ^eocod^^Tycs^eocQeocQ	c^>	co	*_
spno[Qjo jmy o> o o to o <--< o> o o o o go co_____________________________o	o	co cd	o o	o	1
.
•	•TTTATT1CT TA TTT 00	rHQOOOOeQi^ CO ft . O r-j Q* #	#	. fl) rH #	. Oi 03 CO CQ 0^	CO
-UlOJUnjO “I	o © CD Ci Ci C) cr’o o o o ci © © O O O CD o o o o © o o o ci
►J __________I co C9 d C3 CO CO	J73/M 03 CO CO CO CO CO CD CD CO CO 03 CO 00 CO CO CO CO CO CO <73
<	oo uo	©	o ®	?- o I'-	F*
fl	‘DO	>—<	C)C0F'-C0O3	70
•UTOHJO’UI 70 -»	GO	r-	O3»OU0Tf<CO	Q3
__________________• •___________:_____:__________	-4	•	•	___________>d
__________________________________________________________________________________•^al^^CCP^UPCOtTCOOPOrHCT W^UP^^OOOPOgTglOP^UP^^gP^O
				

